# Characterization of the Effect of Hollow Glass Beads on the Mechanical Properties of Structural Adhesives

**DOI:** 10.3390/ma15113817

**Published:** 2022-05-27

**Authors:** João P. J. R. Santos, Daniel S. Correia, Eduardo A. S. Marques, Ricardo J. C. Carbas, Frida Gilbert, Lucas F. M. da Silva

**Affiliations:** 1 Institute of Science and Innovation in Mechanical and Industrial Engineering (INEGI), Campus da FEUP, Rua Dr. Roberto Frias, 400, 4200-465 Porto, Portugal; joao10joanes@gmail.com (J.P.J.R.S.); dcorreia@inegi.up.pt (D.S.C.); carbas@fe.up.pt (R.J.C.C.); 2 ArcelorMittal Global R&D, 60160 Montataire, France; frida.gilbert@arcelormittal.com; 3 Department of Mechanical Engineering, Faculty of Engineering (FEUP), University of Porto, 4200-465 Porto, Portugal; lucas@fe.up.pt

**Keywords:** structural adhesives, hollow glass beads, characterization, scanning electron microscopy, rule of mixtures

## Abstract

Adhesives are extensively used in the automotive and aeronautical industries as they enable the creation of durable and light weight joints, with exceptional strength to weight ratios. The constant search for the means of adapting the mechanical performance of adhesives to each application has led to the use of several types of fillers to change their properties. Following a study on the effect of inorganic fillers, i.e., hollow glass beads, in the failure mechanisms of single lap joint’s (SLJ), this work focuses on the response of the strength and fracture properties of structural adhesives to this filler. To this end, their tensile strength and mode I fracture properties were thoroughly analyzed by performing bulk tensile and double-cantilever beam (DCB) tests, at a quasi-static speed. The specimens were manufactured by adding different %v/v of filler to two epoxy-based crash resistant adhesives. Both adhesives have shown a negligible effect on the tensile strength, a decrease in strain at failure and critical energy release rate in mode I, as well as an increase of the Young’s modulus, for higher % in volume of hollow glass beads. These phenomena were further analyzed recurring to scanning electron microscopy, and the concept of rule of mixtures.

## 1. Introduction

The growing use of structural adhesive joints, particularly in highly technological applications, created a demand for methods to improve their mechanical behavior, tailoring the properties to the application and failure mode, promoting cohesive failure over adhesive failure. Amongst others, some of the methods that have been studied to ameliorate their performance are the combination between adhesive joints with other conventional joining methods, i.e., hybrid joints, changes in the adherends’ surface roughness, as an attempt to improve the adhesion of the joints; and the search for an optimum thickness of the adhesive layer [1].

The addition of micro and nanoparticles as fillers is often a simpler and also more affordable way to change the adhesive’s performance. In general terms, this method results in a composite material, as it synergistically combines two or more materials [1], being able to increase or diminish the response of the material to a certain stimulus depending on the amount and type of particles used. Several examples can be seen in the literature: in terms of thermal or electrical conductivity by adding metallic particles [2,3] or inorganic particles [4,5,6,7,8] it is possible to create conductor materials [2,3,4,5] or insulating materials [6,7,8]; certain fillers can improve the mechanical performance of an adhesive joint [9,10,11] without any relevant side effect up until specific content percentages; by adding rubber particles [12], nowadays widely used in commercial crash resistant adhesives, it is possible to improve the energy absorption capabilities of these materials; the use of hollow glass beads can improve thermal insulation and fire retardation of isolating foams [6,8] or fabrics [7]; amongst many other possible scenarios and outcomes of adhesive doping.

**Figure 1:**
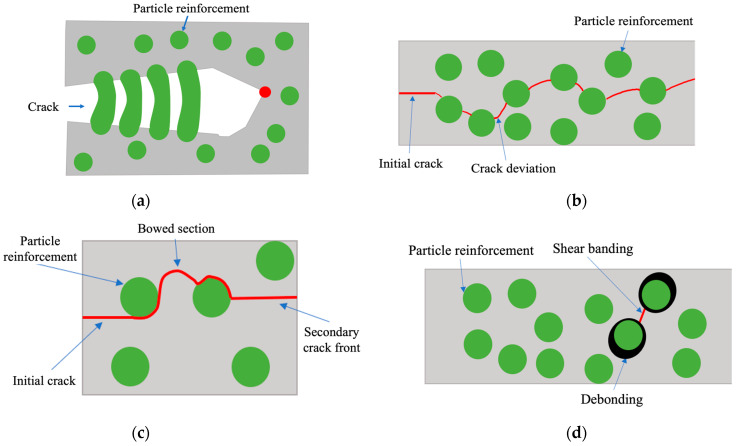
Schematic representation of different toughening mechanisms in particle reinforced polymers. (**a**) Bridging. (**b**) Crack growth deviation. (**c**) Crack pinning and bowing. (**d**) Shear banding due to particle debonding.

The addition of particles as fillers can also significantly influence the fracture mechanics of the adhesives, since they can change the way cracks propagate along the adhesive layer through several damaging or toughening mechanisms, as shown in Figure 1. The bridging mechanism, shown in Figure 1a, can work as a way of enhancing the mechanical properties of the adhesive, and it is highly dependent on the size and geometry of the reinforcements being introduced [1,13]. Crack deviation, shown in Figure 1b, is a mechanism which occurs as a consequence of the presence of particles in the adhesive’s matrix that stop the crack’s progression and force it to change its regular path [1].

Moreover, crack pinning and bowing mechanisms (Figure 1c) are more relevant for brittle materials. Here, changes in the velocity and shape of the crack propagation near the second-phase particles lead to an energy dissipation along the adhesive [1,14]. Microcracking appears as the formation of multiple cracks ahead of the main crack tip, leading to a decrease in the modulus and stress concentrations near them, culminating in an improvement of the adhesive’s fracture toughness [1]. The shear banding mechanism (Figure 1d) derives from local stress concentrations created by the reinforcements and can be provoked by particle debonding and void nucleation. This decreases the stresses next to the particles and facilitates the propagation of the shear bands [1].

Another common damage mechanism is particle debonding and subsequent plastic void growth. In this case, particle debonding occurs due to local stress concentrations at the particle–matrix interfaces. Then, local plastic void growth may appear as a consequence of the previous conditions. This leads to an increase in the dissipated energy, due to the existence of plastic voids [1]. Finally, stress fields around the introduced fillers, e.g., spherical rubber particles, are also reported as a damage mechanism that introduces local stress concentrations around the equator of the particle. For the case of the rubber particles, since they have the capability of bearing loads while functioning as a stress concentrator, they act as an effective toughening mechanism [12,15].

Kinloch et al. [9] investigated how the addition of nanosilica particles to a rubber toughened adhesive influenced its toughness. These particles, which presented an average diameter of 20 nm, led to improvements not only in terms of toughness, but also of single overlap shear strength for amounts between 1% and 8% in weight. The presence of these spheres generated interactions between the stress field around them and the crack tip, furthering the plastic deformation of the adhesive.

Barbosa et al. [10] studied the effect of cork particles on the strength of single lap joints bonded with a structural adhesive. Different amounts of cork (between 0.5% and 5%, in weight) were added to the adhesive layer. The experimental results proved that, for 1% of cork particles, the adhesive showed an increase in terms of ductility and a higher joint strength. Increasing the number of particles up to 2% negatively affected the mechanical behavior, reducing the adhesive toughness.

Forte et al. [16] compared the different energy release rates in mode I for different volumes of fiberglass fibers in an epoxy resin. The introduction of glass fibers to the adhesive layer led to a general decrease of the energy release rate when compared to the neat resin. Analyses performed through scanning electron microscopy (SEM) demonstrated the existence of fiber breakage for every combination of adhesive and fiberglass, and a matrix interface failure in most of the analyzed specimens.

Kishi et al. [11] compared the differences between using polyamide-12 particles and core-shell rubber particles as reinforcements for an epoxy resin by analyzing their impact on the adhesive’s properties. The results showed that the T-peel adhesive strength was improved by increasing the amount of polyamide-12, and the use of core-shell rubber particles as reinforcements improved the adhesive’s strength until a plateau was attained.

Looking more closely at the use of hollow glass beads, few application scenarios use them as structural adhesive fillers; in most cases, their good strength to weight ratio and thermal insulating capabilities are explored to create low thermal conductivity composite or foams, as seen previously. Nevertheless, other applications are possible, as studied by Santos et al. [17], where the hollow glass beads work as low weight weak links inside an adhesive joint to improve their failure reliability, i.e., cohesive failure instead of adhesive failure. This was done by investigating the effect of different percentages of hollow glass beads in the failure mechanisms of single lap joints (SLJ). Results showed that these particles were able to change the joint failure from adhesive to cohesive without any drastic drop in mechanical performance. As such, the hollow spheres worked as weak links inside the adhesive joint. When compared with both solid glass beads and the adhesive—presenting both lower density and lower strength —they actually reduced the weight of the joint, while improving its failure performance.

That said, this work focuses on the tensile strength and mode I toughness response of structural adhesives to the addition of hollow glass beads. To do so, both tensile properties and fracture energy in mode I were determined by performing bulk tensile and double-cantilever beam (DCB) tests, with different configurations in terms of the percentage in volume of glass beads mixed into the adhesives. Subsequently, the studied properties were defined as a function of the %v/v of filler. Additionally, SEM analyses were conducted on the fracture surfaces of the bulk specimens, allowing us to survey the state and distribution of the hollow glass beads, as well as the damage mechanisms that these might have triggered.

To conclude, the concept of Rule of Mixtures (ROM) was used at the end of this study to access its validity as a simple predictive tool to estimate the mechanical properties of any adhesive as a function of %v/v of hollow glass beads. As such, two adhesives were tested through the previously described procedure. One of them was analyzed more extensively, serving as the reference sample, to attain the predicted properties of the glass beads and the second as the benchmark of this theory using the glass bead properties obtained through the first adhesive. If valid, this concept would reduce the need for extensive experimental characterization work in order to simulate through numerical models the behavior of any other doped adhesive joint.

## 2. Experimental Details

In this section, a detailed description of both the materials used, as well as the manufacturing and testing procedures, is provided.

### 2.1. Materials

Two epoxy adhesives were used in this study, both of which were doped with an inorganic filler, i.e., hollow glass beads.

#### 2.1.1. Adhesives

The adhesives studied throughout this work were referred to as Adhesive A and Adhesive B. Both are one component heat cured crash-resistant epoxy-based adhesives, whose aim is to increase durability, crash performance and body stiffness of the components in which they are used. Their main properties are present in Table 1.

The cure cycle used for both adhesives was the same consisting of an isothermal stage at a temperature of 180 °C for 30 min.

#### 2.1.2. Fillers

The filler used in this work consists of thin-walled/hollow glass beads made of soda-lime-borosilicate glass with a white powder appearance. Throughout this work they were called solely hollow glass beads or GBs in short format, but are referred to by the manufacturer (3M Minnisota, Saint Paul, MA, USA) as K37 glass bubbles [18].

They can vary in terms of size and density but are known to be lightweight—being approximately seven times lighter than solid glass—resulting in a high strength-to-density ratio by having a considerable isostatic compressive strength, *σ_c_*, [19].

Table 2 lists the main properties of the hollow glass beads used to dope the adhesives under study.

It is important to state that Adhesive A in its commercialized formulation, i.e., the *as supplied* state, already integrates 5%, in volume, of the hollow glass beads used in this study. Nevertheless, this fact was not considered throughout the experimental study, only being applied to the rule of mixtures analysis. As for Adhesive B, prior to manufacturing, no glass filler was known to be present in its composition.

### 2.2. Specimen Manufacturing

All specimens were manufactured recurring to procedures detailed by Banea et al. [20], e.g., the appropriate molds to use, proper pressure conditions, among other factors. A hot plate press was used as the cure setup, ensuring, through conduction heating, the cure cycle previously detailed, as well as 2 MPa of pressure.

This work analyzed different adhesive configurations in the *as supplied* and *doped* states, the filler being the previously detailed hollow glass beads. As a baseline, the %v/v’s used will never surpass the 20% limit since the use of fillers is usually set for small percentages and much higher values would make the filler become the matrix, turning it into a foam instead of a doped adhesive.

**Figure 2:**
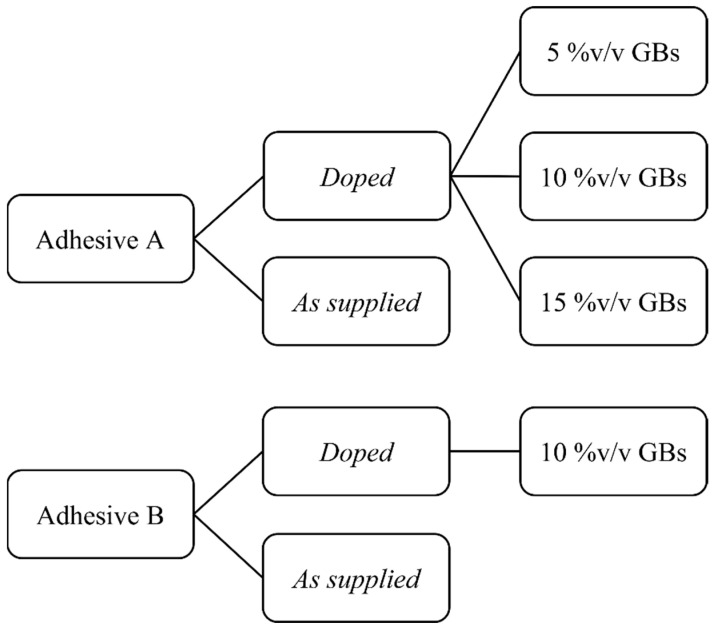
Schematic diagram of the adhesive configurations analyzed for both adhesives (A and B) in the *as supplied* and *doped* states.

The manufacturing plan followed two main premises: the %v/v’s used in a previous study on SLJ failure mechanisms [17], as well as the need for a reference and a benchmark subject for the study on the validity of property prediction through ROM. As such, the study was divided into an extensive characterization of Adhesive A with small 5%v/v GB increments from 0% to 15%, used as the reference data sample and a less considerable one for Adhesive B with two different configurations at 0% and 10%, as the benchmark data sample. This said, to clarify the specimen manufacturing plan a schematic representation is presented in Figure 2.

The incorporation of the hollow glass beads in the polymer matrix was carried out by mixing them directly with the epoxy adhesive. This process was performed after a preheating of the adhesive, in order to reduce their viscosity and facilitate the distribution of the particles, since both adhesives were highly viscous. A centrifuge mixing machine, Hauschild SpeedMixer^®^ DAC 150 (Hamm, Germany), was used in this process. In each batch, it operated for 40 s, increasing gradually from 500 rpm to 3000 rpm in the first 10 s and then maintaining the rotational speed at 3000 rpm for the remaining time.

#### 2.2.1. Tensile Specimens

Bulk specimens were obtained by CNC machining 2 mm thick cured adhesive plates manufactured in a process conforming with the NF T 76-142 standard [21]. This manufacturing process involves the use of a steel mold to press the adhesive in the uncured state, and a silicone rubber frame that delimits the adhesive on its sides by applying hydrostatic pressure to it, while inside the hot plate press. By doing so, an acceptable surface finish was assured, as well as preventing the appearance of voids. The adhesive was cured by applying the previously described temperature and pressure conditions.

**Figure 3:**
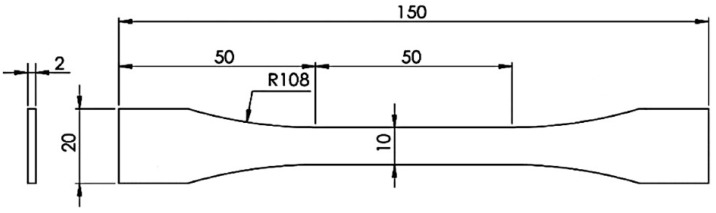
Representation of tensile specimens’ geometry, adapted from BS 2782 [22], dimensions in mm.

Finally, the specimens were machined according to the British standard, BS 2782 [22], with the geometrical specifications presented in Figure 3.

#### 2.2.2. DCB Specimens

**Figure 4:**
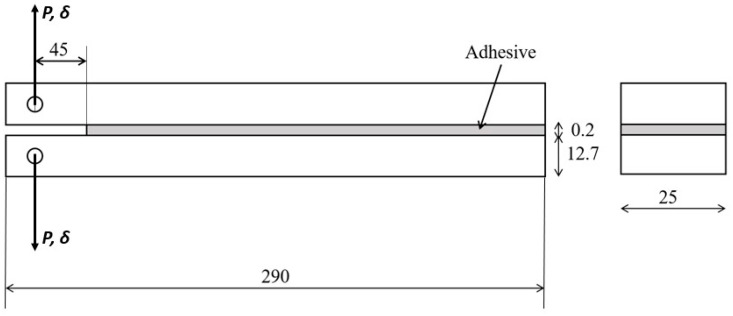
Representation of DCB specimens’ geometry and testing, dimensions in mm.

DCB specimens were manufactured as shown in Figure 4.

The substrates used were made of high strength steel—DIN 40CrMnMo7—to prevent plastic deformation during the test. These were CNC machined, with general tolerances of 0.2 mm, grit blasted with alumina grit at 0.6 MPa of pressure to remove oxides and create roughness in the bonded area and were finally degreased with acetone to ensure good adhesion.

A pre-crack, 45 mm long, was created by placing a sharp razor blade with 0.1 mm of thickness in one end of the bonded area, improving the fracture behavior by facilitating cohesive failure. A calibrated tape of 0.2 mm was positioned at the other end of the substrates to ensure the adhesive thickness of 0.2 mm. The adhesive was then applied and was cured with the prescribed temperature and pressure.

### 2.3. Testing Procedures

Both the tensile and the DCB tests were carried out in an INSTRON^®^ 3367 universal testing machine (Norwood, MA, USA), equipped with a load cell of 30 kN. Both tests were performed at quasi-static speeds of 1 mm·min^−1^ and 0.2 mm·min^−1^, respectively. Three specimens were tested for each adhesive configuration in both test types, their results being properly treated and validated as acceptable to assure statistical relevance.

#### 2.3.1. Tensile Tests

Bulk tensile tests were fulfilled to assess the effect of adding hollow glass beads to the adhesive’s properties. This was done by evaluating changes on the Young’s modulus, *E*, maximum tensile strength, *σ_f_*, and strain at failure, *ε_f_*, for different %v/v of filler.

The tests were conducted at 1 mm·min^−1^ and the displacement measured recurring to an INSTRON^®^ 2630-107 static clip-on extensometer (Norwood, MA, USA), with 25 mm of gauge length measuring up to 100% strain.

**Figure 5:**
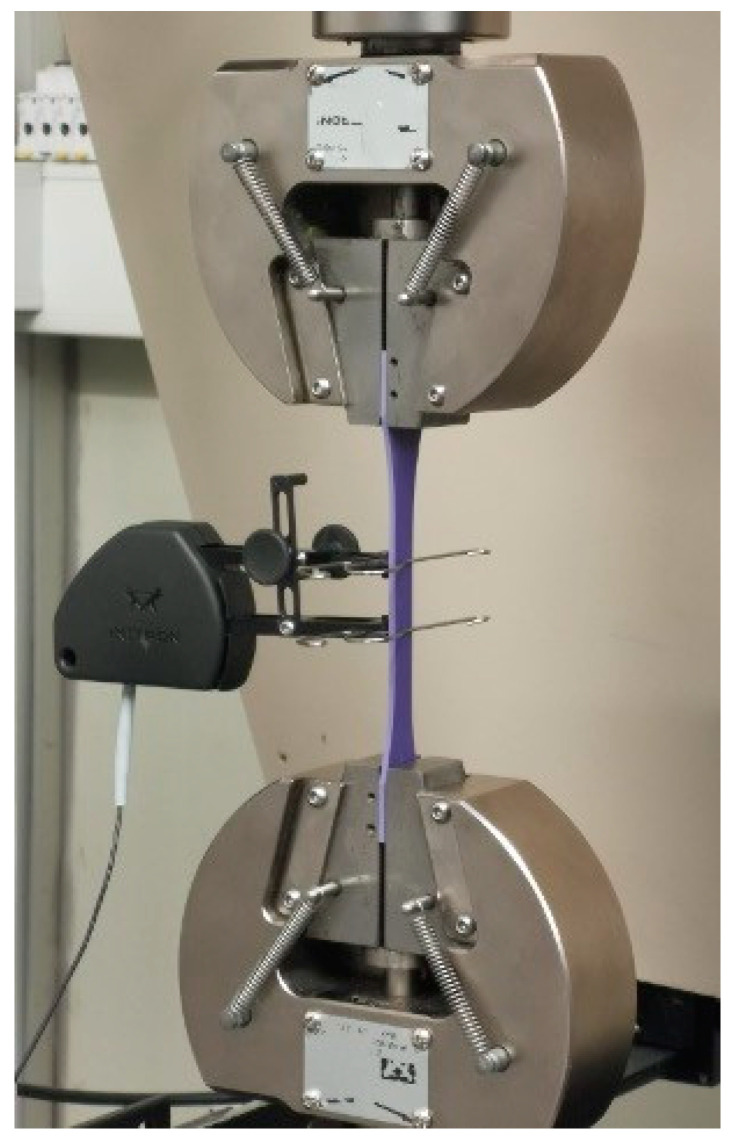
Tensile test setup. The specimen is fixed with wedge action tensile grips and a clip-on extensometer is used to record the test’s extension.

For each test, the load-displacement (*P* − *δ*) curve was registered up until failure, and its respective stress–strain curve was calculated, from which all the previously detailed properties were extracted. The test setup used was as depicted in Figure 5.

#### 2.3.2. DCB Tests

DCB tests were performed with the purpose of analyzing the behavior of the critical energy release rate in mode I, *G_IC_*, when adding hollow glass beads to the adhesive’s matrix.

Prior to testing, all specimens were loaded in mode I at low load levels to open a sharp pre-crack and ensure a stable crack propagation, for which a new initial crack length, *a*_0_, was recorded.

**Figure 6:**
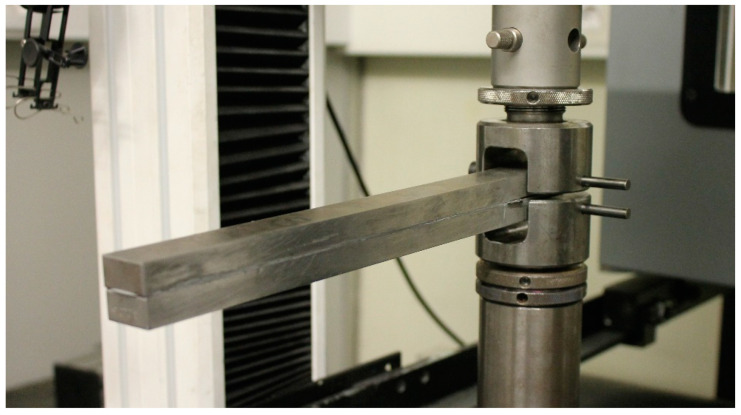
DCB test setup. The specimen is fixed with U-shaped pin-based systems.

The tests were conducted at 0.2 mm·min^−1^, and each load displacement (*P* − *δ*) curve was registered. The test setup used was as presented in Figure 6.

The compliance-based beam method (CBBM) data reduction scheme was used for the determination of the energy release rate in mode I, this method being mainly developed by Moura et al. [23].

This technique allows us to determine the crack length from the specimen’s compliance, without requiring us to directly measure it. Additionally, it considers the fracture process zone (FPZ) effects in the calculation of the fracture energy [23]. Since this area is defined by the existence of multiple micro-cracks and a plasticized zone ahead of the major crack, that also absorbs energy [23], it needs to be considered in the data reduction formulation.

As a result, using the CBBM methodology [23], Equation (1) is used to calculate the fracture energy in mode I:

(1) $$G_{I}=\frac{6P^{2}}{b^{2}h}\left(\frac{2a_{eq}^{2}}{h^{2}E_{f}}+\frac{1}{5G_{13}}\right),$$

where *P* represents the load, *b* the specimen width, *h* the substrate thickness, *G*_13_ the steel’s shear modulus, *a_eq_* the equivalent crack’s length, and *E_f_* the corrected flexural modulus. This last parameter is used to account for the substrates’ rotation near the crack tip [23].

As previously said, the equivalent crack length, *a_eq_*, is calculated based on the specimen’s compliance [23], using a root rotation correction for determining the initial crack length and another to take into account the fracture process zone [24].

### 2.4. Scanning Electron Microscopy Analysis

Scanning electron microscope (SEM) analyses were performed utilizing a JEOL JSM 6301F/Oxford INCA Energy 350/Gatan Alto 2500 microscope (Tokyo, Japan). The use of this equipment allowed us to determine the level of damage in the glass spheres, their diameter range, the distribution of the beads along the adhesive, and the fracture modes. The X-ray diffraction analysis associated with this equipment also enabled us to precisely determine the chemical nature of the materials being tested.

## 3. Experimental Results and Discussion

As stated previously, the tensile and DCB tests were performed for different amounts of hollow glass microspheres, measured in volume fractions. The bulk specimen fracture surfaces were analyzed via SEM to understand the adhesives composition, damage mechanisms, and the hollow glass beads’ size and shape, among other factors.

The results were first presented for each adhesive separately, concluding with a summary discussion of the behaviors found.

### 3.1. Results

#### 3.1.1. Adhesive A

Adhesive A was extensively tested in four different configurations, as supplied and doped with 5%v/v, 10%v/v and 15%v/v of GBs.

##### Tensile Tests

**Figure 7:**
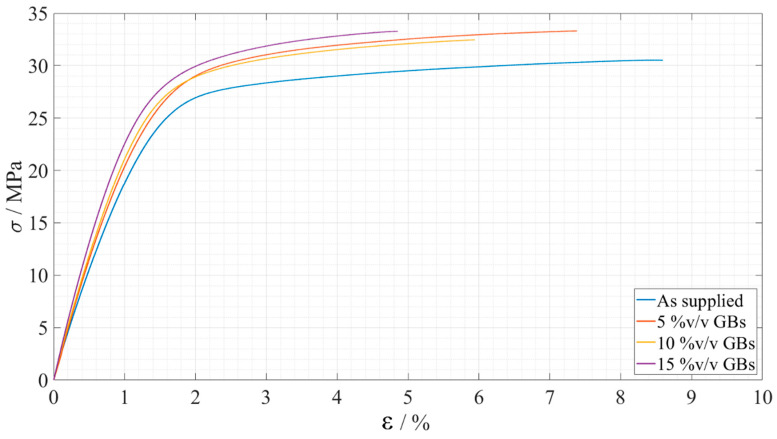
Representative stress-strain curves of the bulk tensile tests for each Adhesive A configuration. As supplied in presented in blue, 5%v/v GBs in orange, 10%v/v GBs in yellow, and 15%v/v GBs in purple.

The stress, *σ*, strain, *ε*, curves obtained for the tensile tests showed the tendencies present in Figure 7, where one representative curve for each configuration is shown.

The mean values of the Young’s modulus, *E*, maximum tensile stress, *σ_f_*, and strain at failure, *ε_f_*, can be seen at Table 3.

##### DCB Tests

**Figure 8:**
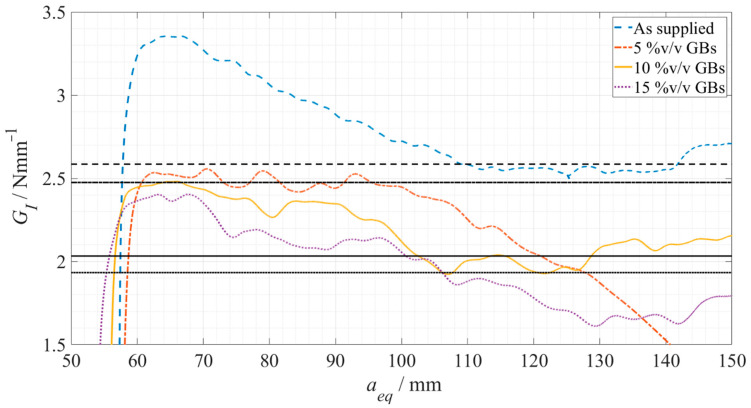
Representative R-curves for each Adhesive A configuration. As supplied is presented in a dashed blue line, 5%v/v GBs in a dot-dashed orange line, 10%v/v GBs in a full yellow line, and 15%v/v GBs in a dotted purple line, being each G_IC_ plateau represented in dark grey with the corresponding line pattern.

Based on the experimental *P*-*δ* curves, and using the CBBM methodology, the respective R-curves were determined, and consequently the critical energy release rates in mode I were obtained. These R-curves are represented in Figure 8, for Adhesive A, in the *as supplied* state and *doped* state, with 5%, 10% and 15% volume of glass beads, respectively.

The results obtained regarding the critical energy release rate in mode I can be seen in both Figure 8, corresponding to each respective horizontal line, and as a summary in Table 4.

##### SEM Analysis

**Figure 9:**
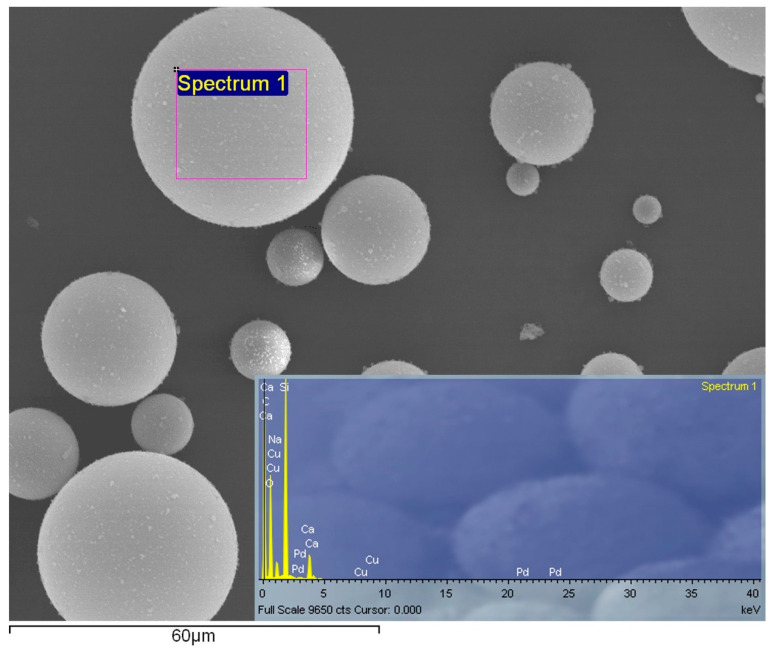
Backscatter electron analysis of the glass microspheres, and the respective spectrum analysis of the spheres in the highlighted area (Spectrum 1).

With recourse to SEM, the hollow spheres were analyzed, Figure 9 shows an external rounded shape and a range of diameter values that vary between 6 and 60 µm.

A spectrum analysis of the hollow glass beads demonstrated that these are mainly composed of silicate, sodium and calcium. Moreover, the particles presented a coating composed of copper and palladium.

After testing the bulk specimens, their fracture surfaces were evaluated through SEM.

**Figure 10:**
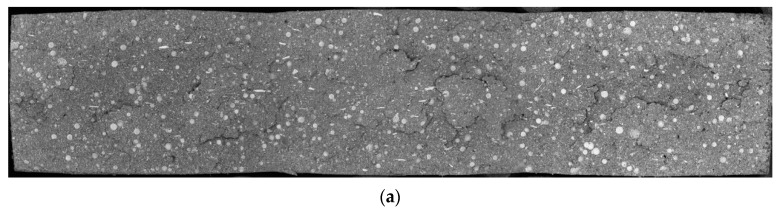

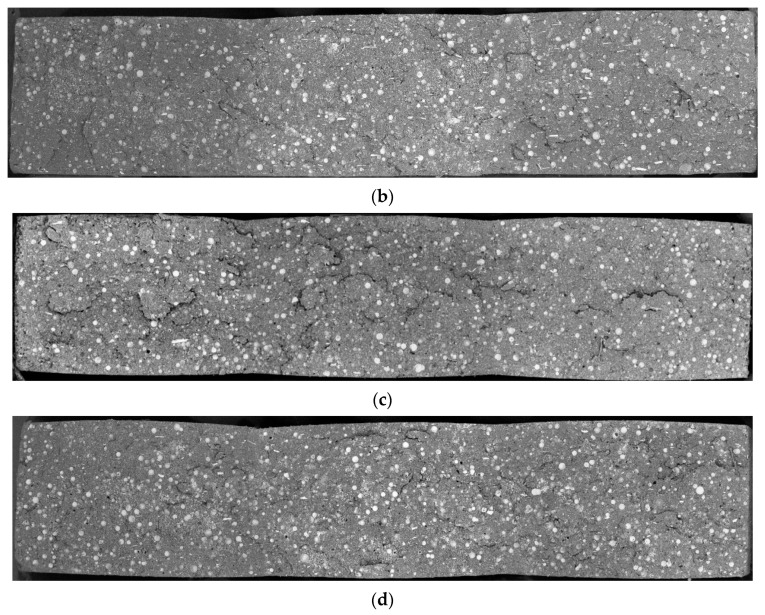
Fracture surfaces of the bulk specimens of Adhesive A, obtained through backscatter electron analysis. (**a**) Adhesive A *as supplied*. (**b**) Adhesive A *doped* with 5% GBs. (**c**) Adhesive A *doped* with 10% GBs. (**d**) Adhesive A *doped* with 15% GBs.

One specimen of each adhesive configuration analyzed is shown in Figure 10.

Throughout the fracture surfaces of the adhesive, in all configurations, it was possible to see a uniform sphere distribution, confirming that the method used to mix the glass beads works properly, as expected. Figure 10a also confirms that Adhesive A already had glass beads in its supplied formulation.

**Figure 11:**
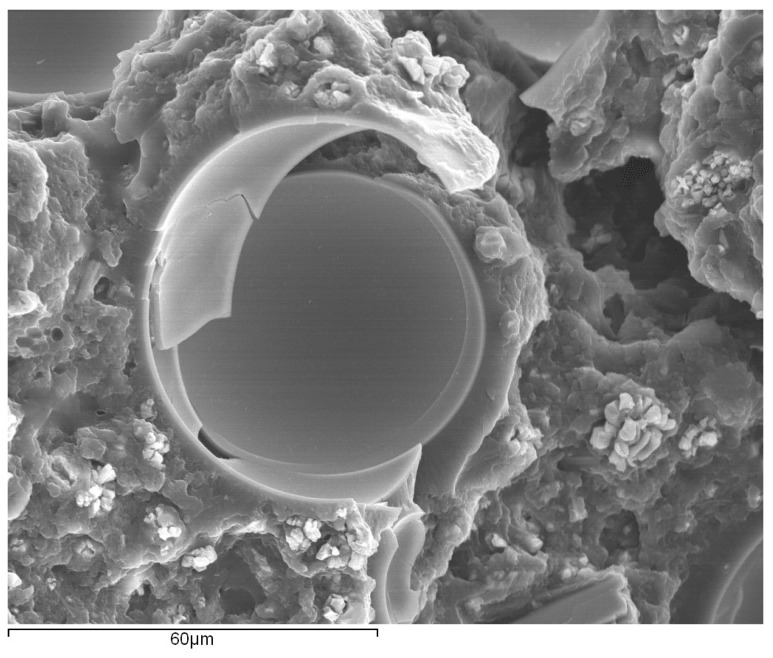
Fracture surface of a bulk specimen with focus on the region around the glass beads, obtained through secondary electron analysis.

A careful observation of the regions near the glass particles, as shown in Figure 11, shows a very good level of bonding between them and the adhesive’s matrix. Additionally, some of the glass microspheres were found to be completely shattered, and there was no evidence of a particular damage mechanism taking place as a consequence of their presence.

#### 3.1.2. Adhesive B

Adhesive B was simply tested in two different configurations: *as supplied* and *doped* with 10%v/v of GBs.

##### Tensile Tests

**Figure 12:**
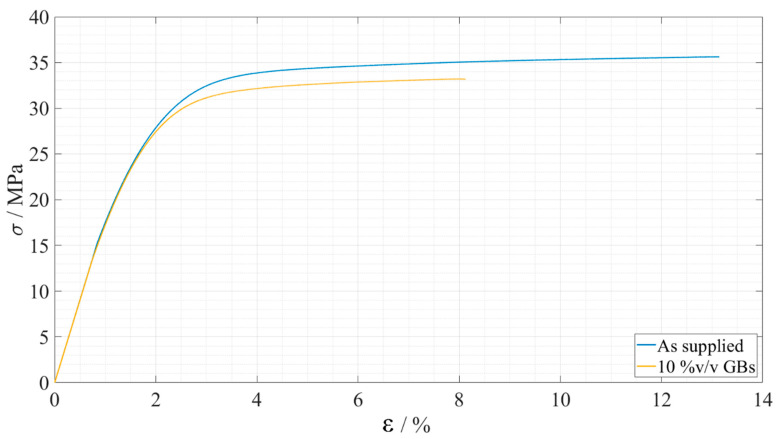
Representative stress-strain curves of the bulk tensile tests for each Adhesive B configuration. As supplied is presented in blue and 10%v/v GBs in yellow.

For Adhesive B, one of the stress, *σ*, and strain, *ε*, curves for each configurations is shown in Figure 12.

A summary of the evolution of the Young’s modulus, *E*, as well as the maximum tensile stress, *σ_f_*, and the strain at failure, *ε_f_*, can be observed in Table 5

##### DCB Tests

**Figure 13:**
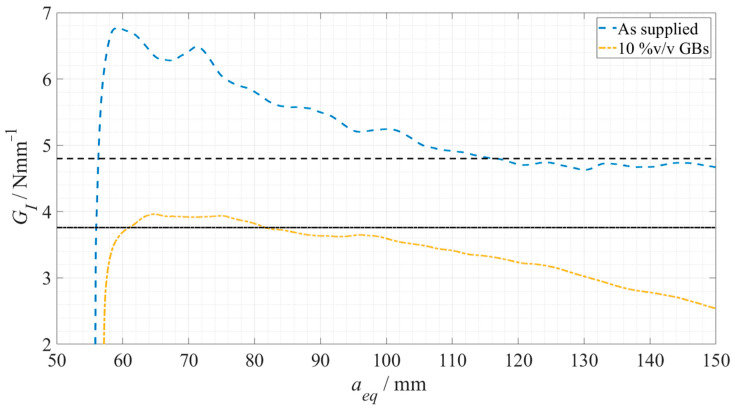
Representative R-curves for each Adhesive B configuration. As supplied is presented in a dashed blue line and 10%v/v GBs in a dot-dashed yellow line, each G_IC_ plateau being represented in dark grey with the corresponding line pattern.

Once more, CBBM was used to determine the corresponding R-curves and the critical energy release rate in mode I, as presented in Figure 13.

The results obtained regarding the critical energy release rate in mode I for this adhesive are represented by the respective horizontal lines in Figure 13 and are summarized in Table 6.

##### SEM Analysis

**Figure 14:**
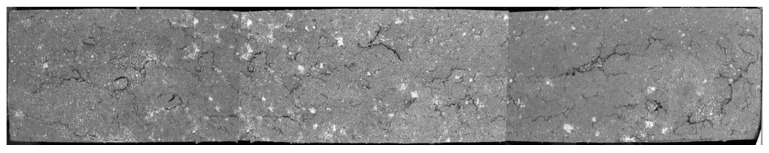
Fracture surfaces of bulk specimens of Adhesive B in the *as supplied* state, obtained through backscatter electron analysis.

For Adhesive A the spheres’ behavior was well defined, Adhesive B was solely analyzed in the *as supplied* state, a representative fracture surface is presented in Figure 14.

**Figure 15:**
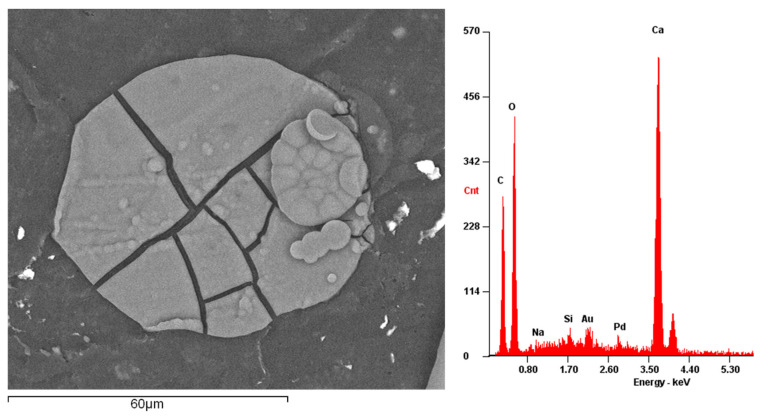
Fracture surface of a bulk specimen of Adhesive B, with focus on the region around a broken calcium carbonate particle, obtained through secondary electron analysis (**left**) and its respective spectrum analysis (**right**).

Using a higher magnification level, it was possible to notice the presence of unknown fillers in the supplied state, uniformly spread along the adhesive’s matrix. An analysis of the composition of these particles found them to be composed of calcium carbonate (Figure 15), a common mineral filler used in polymers [25].

Looking carefully to the regions near the calcium carbonate, a few were found to be broken but there was no evidence of a particular damage mechanism taking place as a consequence of their presence.

### 3.2. Summary and Discussion

**Figure 16:**
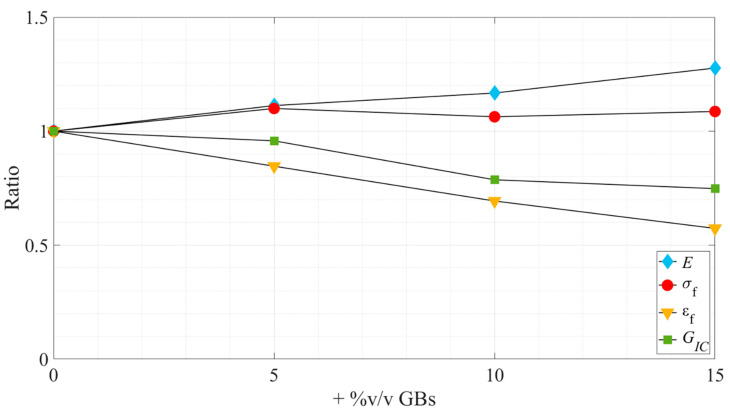
Variation, in ratio related to the *as supplied* state, of the tensile and fracture properties as a function of the added %v/v GBs—Adhesive A.

**Figure 17:**
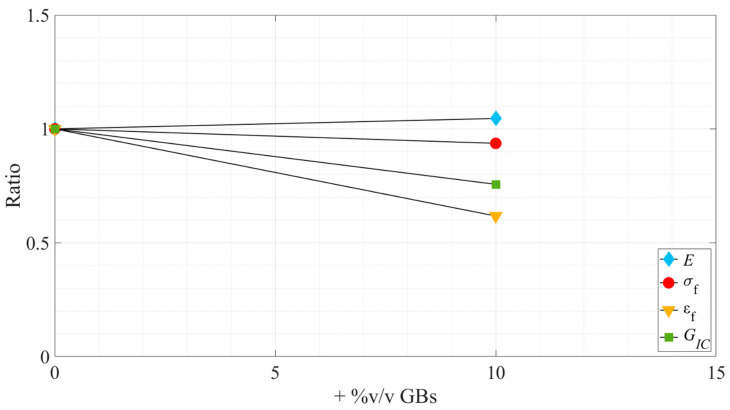
Variation, in ratio related to the *as supplied* state, of the tensile and fracture properties as a function of the added %v/v GBs—Adhesive B.

First, it is possible to see, both in the experimental curves and when analyzing consecutive values obtained for each propriety, that they did not always show significant variations between them due to the reduced effect of the hollow glass beads in the evaluated properties. Therefore, to summarize all the results in a clearer way, Figure 16 and Figure 17 show the tendencies followed by the studied properties as a function of the glass particle content, for Adhesive A and Adhesive B, respectively. In addition, these clear trends can also be seen in Section 4, where the Young’s Modulus and the critical energy release rate in mode I are studied.

Looking at the bigger picture, summarized in Figure 16 and Figure 17, it demonstrates that the introduction of hollow glass beads into the adhesive has similar effects on both adhesives under study.

Overall, the influence of the microspheres on the tensile strength was found to be residual, as in both adhesives it did not show any statistical trend oscillating slightly near the *as supplied* state values. On the other hand, the strain at failure and the Young’s modulus were clearly influenced by it, the first being subjected to a gradual decrease, while the second was subjected to a gradual increase. Therefore, it can be stated that the addition of hollow glass beads to the adhesive makes it more brittle; this statement is supported by the reduction in the area below the stress–strain curves. It is also supported by the reduction of the ductile/tough adhesive load supporting area as more GBs are introduced in the matrix resulting in more local rigid/brittle load bearing zones that turn the overall material more rigid and brittle.

With regard to the fracture toughness, similar to what was reported in the work of Forte et al. [17] regarding the influence of fiber-glass fibers in the adhesive layer, there is an overall decrease of the critical energy release rate in mode-I. This behavior translates into a decreasing capability of the adhesive to absorb energy as the amount of glass particles increases, resulting in the conclusion that this filler does not work as a toughening mechanism. Instead, they weaken the adhesive since they appear to be in a severely damaged state, as seen in Figure 11, which is a consequence of their brittleness and lower strength. They have no capacity for absorbing energy and do not induce toughening mechanisms, as the ones described previously in the introductory section.

Finally, it can generally be said that the addition of hollow glass beads to structural adhesives worked as a weakening process rather than a reinforcement. Furthermore, instead of inducing a toughening mechanism, it reduced the adhesive’s capacity for absorbing energy, turning it into a more brittle material. Nevertheless, their presence contributes to the generation of stress concentrations, representing weak links in the adhesive’s matrix that change the crack propagation path to the interior of the adhesive, preventing adhesive failure with only a small penalty in the mechanical properties. This phenomenon was seen by Santos et al. [1], by promoting a safer, more predictable failure mode of SLJ specimens, i.e., cohesive failure.

## 4. ROM Validity Analysis

The concept of Rule of Mixtures (ROM) was used in this study to access its validity as a simple predictive tool to estimate the effective mechanical properties of the adhesive as a function of the filler content, reducing the need for extensive experimental characterization work when using other adhesives, and also as a way to estimate the properties of the hollow glass beads [26].

As stated previously, Adhesive A was used as a reference sample, with four data points, to define a correct fit between the rule of mixtures and the experimental data, obtaining a more accurate prediction of the properties of the GBs.

Adhesive B was used as the benchmark sample, with only two data points. Then the previously determined properties of the glass beads were recurred to establish the predictive rule of mixtures and its adequacy was compared to the experimental data of Adhesive B. With this in mind, ROM was applied to the Young’s modulus and mode I fracture toughness of both adhesives, comparing the results extracted for the glass beads’ properties in each case, in order to understand the applicability of this theory.

At this point it is important to remember that Adhesive A in its commercialized formulation, i.e., the *as supplied* state, already integrates 5% in volume of the hollow glass beads used in this study, and Adhesive B has no glass filler in its composition.

### 4.1. Young’s Modulus

To apply the rule of mixtures to the Young’s modulus, the Reuss model was used [27], which is ruled by Equation (2), adapted for this case:

(2) $$E={\left(\%v/v\right)}_{Adh}\cdot E_{Adh}+{\left(\%v/v\right)}_{GB}\cdot E_{GB},$$

where (%v/v)*_Adh_*and (%v/v)*_GB_* are the volume fractions of the adhesive with 0%v/v of GBs and the hollow glass beads themselves; as well as *E_Adh_* and *E_GB_* being the Young’s modulus and the same materials, respectively.

#### 4.1.1. Implementation

To ensure the best correlation between the experimental Young’s modulus and the ROM, defined by its extreme values—*E_Adh_* and *E_GB_*—, the rule was fitted to the obtained data points considering upper and lower threshold lines. These boundaries take the values of plus and minus two times the mean standard deviation (± 2·$\bar{\sigma }$) of the experimental data points and can be seen in the plots as dark grey dashed lines.

##### Adhesive A

By fitting this model’s equation to the experimental Young’s modulus values of each

**Figure 18:**
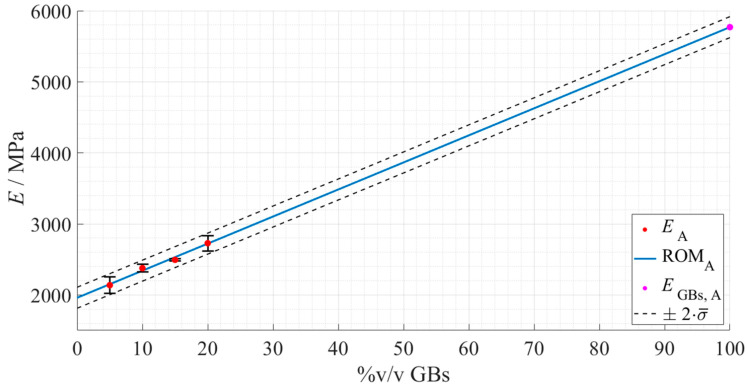
Rule of mixture’s prediction for the Young’s modulus evolution, with regard to Adhesive A as a function of the true %v/v of hollow glass beads. The experimental data points are presented in red with the respective standard deviation, the ROM plot in a full blue line, the predicted Young’s modulus of the GBs in magenta, and the standard deviation based fitting limits in dashed dark grey lines.

Adhesive A configuration, the rule represented in Figure 18 was obtained.

Taking into account the fact that Adhesive A in the *as supplied* state, = already contains 5 %v/v of glass particles, the following values extracted for *E_Adh_* and *E_GB_* correspond to the predicted Young’s modulus of Adhesive A without any glass particles within its matrix, and the hollow glass beads themselves (Table 7).

##### Adhesive B

**Figure 19:**
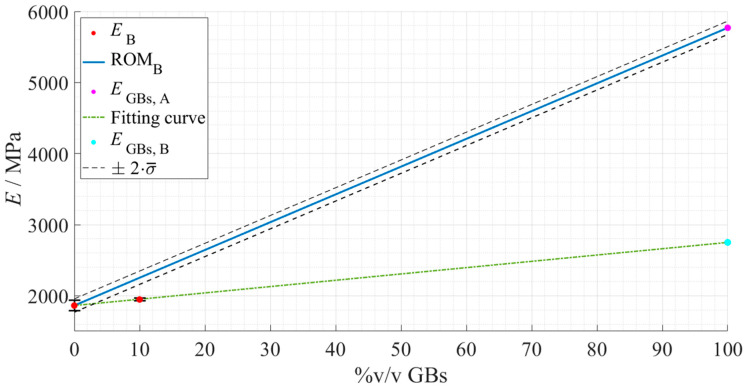
Rule of mixture’s prediction for the Young’s modulus evolution, with regard to Adhesive B as a function of the true %v/v of hollow glass beads. The experimental data points are presented in red with the respective standard deviation, the ROM plot in a full blue line, the previously predicted Young’s modulus of the GBs in magenta, and the standard deviation based fitting limits in dashed dark grey lines. Considering the deviated results, the actual fitting line of the experimental data of Adhesive B is presented in a dot-dashed green line, and a new predicted Young’s modulus of the GBs in cyan for comparison purposes.

Repeating the same procedure for Adhesive B led to the following rule, represented in Figure 19.

Through this model the following values were obtained regarding the Young’s modulus of Adhesive B without glass particles, that is, in the *as supplied* state, and the Young’s modulus of hollow glass beads (Table 8).

#### 4.1.2. ROM Validity

The prediction for the Young’s modulus of Adhesive A, without the 5%v/v of GBs already present in its formulation, was considered reasonable—1960 MPa. This is when taking into account both the reverse effect of the previously detected enhancement of the stiffness by adding hollow glass beads, and the good linear fit of the ROM to the experimental data.

As for the results obtained for the Young’s modulus of the hollow glass beads, to better understand what values were expected, a study conducted by Verweij et al. [28] was analyzed. Focused on the properties of hollow glass bead composites (100% GBs), this work detailed that these particles have lower stiffness than their bulk counterparts, which are usually comparable to the orders of magnitude of organic polymeric materials (10^3^ < *E* < 10^4^ MPa).

For Adhesive A, the exceptional linear correlation with the experimental data estimated that the spheres’ Young’s modulus would be around 5770 MPa, which falls within the expectable values detailed by the previously mentioned study. Nevertheless, this result should be looked at with care, given the uncertain nature of the applicability of this method.

Now, considering Adhesive B, to validate this predictive method, the application of Equation (2) (blue line in Figure 19)—considering the *E_Adh_* of Adhesive B and *E_GB_* attained for Adhesive A—should fit the experimental data of Adhesive B (green line in Figure 19). However, looking to the actual results, one can conclude that, due to the discrepancy between the ROM (in blue) and experimental fitting (in green) lines, this method was not validated. As such, even though the experimental Young’s modulus of Adhesive B showed the same type of behavior—increasing with the addition of a higher %v/v of glass particles—this increase was not as pronounced as the one reported for Adhesive A, i.e., 2810 MPa instead of 5770 MPa.

Several reasons can begin to justify this behavior, the most obvious being the fact that for the second adhesive only two points were tested, creating a straight line between them, which introduces less statistical variability into the analysis. Another possibility is having different degrees of interaction between the glass beads and the constituents of each adhesive type, reducing the spheres’ effect on the stiffness of Adhesive B.

### 4.2. Critical Energy Release Rate in Mode I

For the *G_IC_*, the rule of mixtures used was based on the Voigt model [29], which is associated with Equation (3), rearranged for this case:

(3) $$G_{IC}={\left(\frac{{\left(\%v/v\right)}_{Adh}}{G_{Adh}}+\frac{{\left(\%v/v\right)}_{GB}}{G_{GB}}\right)}^{-1},$$

*G_Adh_* and *G_GB_* being the critical energy release rate in mode I of the adhesive with 0%v/v of GBs and the hollow glass themselves, respectively.

#### 4.2.1. Implementation

Once more, to ensure the best correlation between the experimental *G_IC_* and the ROM, defined by its extreme values—*G_Adh_* and *G_GB_*—the rule was fitted to the obtained data points considering the previously defined range of plus and minus two times the mean standard deviation (± 2·$\bar{\sigma }$) of the experimental data points and can be seen in the plots as dark grey dashed lines.

##### Adhesive A

**Figure 20:**
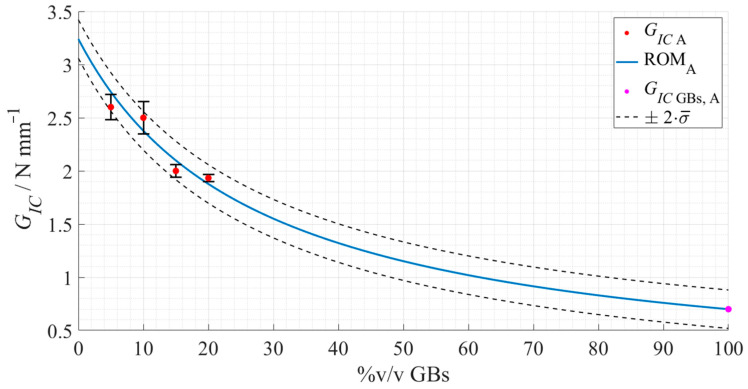
Rule of mixture’s prediction for the *G_IC_* evolution, with regard to Adhesive A as a function of the true %v/v of hollow glass beads. The experimental data points are presented in red with the respective standard deviation, the ROM plot in a full blue line, the predicted mode I fracture toughness of the GBs in magenta, and the standard deviation based fitting limits in dashed dark grey lines.

By fitting this model’s equation to the *G_IC_* of Adhesive A for the different tested configurations, the rule represented in Figure 20 was obtained.

The values for the parameters regarding the *G_IC_* of Adhesive A without glass beads, and the hollow glass beads themselves, *G_Adh_* and *G_GB_*, respectively, were extracted from the ROM and presented in Table 9.

##### Adhesive B

**Figure 21:**
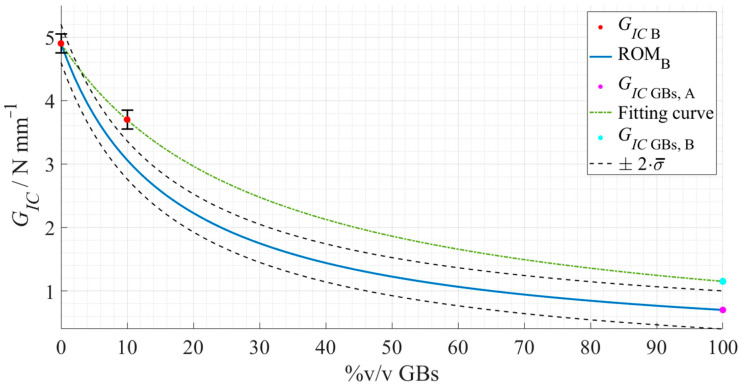
Rule of mixture’s prediction for the *G_IC_* evolution, with regard to Adhesive B as a function of the true %v/v of hollow glass beads. The experimental data points are presented in red with the respective standard deviation, the ROM plot in a full blue line, the previously predicted mode I fracture toughness of the GBs in magenta, and the standard deviation based fitting limits in dashed dark grey lines. Considering the deviated results, the actual fitting line of the experimental data of Adhesive B is presented in a dot-dashed green line, and a new predicted mode I fracture toughness of the GBs in cyan for comparison purposes.

Applying once more the Voigt model, now to Adhesive B, led to the following rule of mixtures, represented in Figure 21.

Having applied this model, the values represented in Table 10 were obtained regarding the *G_IC_* of Adhesive B (*as supplied*), *G_Adh_*, and the hollow glass beads, *G_GB_*.

#### 4.2.2. ROM Validity

The base *G_IC_* estimation for Adhesive A without the intrinsic 5%v/v GBs was considered acceptable—3.2 N·mm^−1^—due to the good correlation between the ROM function used to fit the experimental data, as well as the verification of the opposite behavior to the previously observed fracture toughness weakening when adding glass spheres.

For the mode I fracture toughness of the hollow glass beads, once more, to validate this predictive method, the application of Equation (3) (blue line in Figure 21)—considering the *G_Adh_* of Adhesive B and *G_GB_* attained for Adhesive A (magenta dot in Figure 19)—should fit the experimental data of Adhesive B (green line in Figure 21). Taking this into consideration, the values predicted were consistent with the expected more fragile behavior, but different estimations were presented for each adhesive, and Figure 21 shows the discrepancy between the ROM (in blue) and the experimental fitting (in green) plots.

Nevertheless, a smaller difference was found this time, i.e., from 0.7 N·mm^−1^ to 1.2 N·mm^−1^. This fact might be explained by the type of rule used, inverse instead of linear proportionality, which starts to stabilize for higher volume percentages of GBs, creating less room for such steep differences. The fracture mechanisms involved depend less on the compatibility between the materials since the spheres are mostly damaging the adhesive, reducing its *G_IC_*.

Contrary to the Young’s modulus prediction, this time no helpful information was found in the literature with which to interpret the values obtained. However, the same reasons previously stated could explain, once more, why these differences were observed, e.g., due to the lack of statistical variability for Adhesive B and the possibility of having less compatibility between the materials. The last one is supported by the reduction of the effects of the glass beads in both increasing the Young’s modulus and reducing the fracture toughness as seen in Figure 19 and Figure 21.

## 5. Conclusions

The main aim of this work was to determine how the strength and fracture properties of two structural adhesives vary with the introduction of different amounts of hollow glass particles. For that purpose, bulk tensile tests and fracture tests were carried out to mechanically characterize the adhesives for different filler contents. The tensile tests allowed us to determine the tensile strength, strain at failure, and the Young’s modulus, while the fracture tests in mode I provided the corresponding critical energy release rate. These tests were performed at a quasi-static speed.

The main conclusions which can be extracted from the present work were as follows:

- The tensile strength of the adhesives showed a negligible variation with the varying amount of glass microspheres;
- The strain at failure exhibited a continuous decrease with the growing amount of glass beads;
- The Young’s modulus increased with the increasing volume occupied by the particles;
- The critical energy release rate, *G_IC_*, decreased by increasing the %v/v of hollow glass spheres;
- Hollow glass beads generally acted as weakening agents on the studied adhesives, causing them to become more brittle materials with a reduced capability for absorbing energy;
- The curves obtained by the rule of mixtures were found to work satisfactorily as methods for estimating the mechanical properties as a function of the volume fraction of filler, especially for Adhesive A, leaving a few open questions about Adhesive B.

Finally, with regard to the ROM study, more investigation should be carried out to assess the applicability of these models to estimate the mechanical properties of doped adhesives in general, especially for higher volume percentages of filler (<50%). The consistency of this method must also be analyzed, considering different adhesives, as presented in this work, to understand if any material dependency exists, which remains unclear at this stage.

## Figures and Tables

**Table 1:** Main properties of the structural adhesives used, provided by the supplier.

Property	Adhesive A	Adhesive B
*ρ*/gcm^−3^	1.26	1.18
*E*/MPa	2100	1800
*σ_f_*/MPa	32	35
*ε_f_*/%	≈6	≈10

**Table 2:** Main properties of the hollow glass spheres, provided by the supplier [18].

Property	Hollow Glass Beads
*ρ*/gcm^−3^	0.37
Color	White, powdery
Composition	Soda-lime-borosilicate glass
*d_median_*/µm	45
*σ_c_*/MPa	20.6

**Table 3:** Evolution of the tensile properties’ as a function of %v/v GBs—Adhesive A.

Property	*As Supplied*	5%v/v GBs	10%v/v GBs	15%v/v GBs
*E*/MPa	2136.6 ± 116.9	2376.1 ± 54.2	2493.6 ± 14.9	2727.6 ± 109.5
*σ_f_*/MPa	30.2 ± 0.4	33.2 ± 0.4	32.1 ± 0.4	32.8 ± 0.5
*ε_f_*/%	8.6 ± 0.3	7.2 ± 0.5	6.0 ± 0.3	4.9 ± 0.6

**Table 4:** Evolution of the critical energy release rates in mode I as a function of %v/v GBs—Adhesive A.

Property	*As Supplied*	5%v/v GBs	10%v/v GBs	15%v/v GBs
*G_IC_*/N·mm^−1^	2.6 ± 0.1	2.5 ± 0.2	2.0 ± 0.1	1.9 ± 0.1

**Table 5:** Tensile properties’ evolution as a function of %v/v GBs—Adhesive B.

Property	*As Supplied*	10%v/v GBs
*E*/MPa	1861.5 ± 72.8	1946.8 ± 21.0
*σ_f_*/MPa	35.5 ± 0.2	33.2 ± 0.2
*ε_f_*/%	13.1 ± 0.1	8.0 ± 0.1

**Table 6:** Critical energy release rate in mode I evolution as a function of %v/v GBs—Adhesive B.

Property	*As Supplied*	10%v/v GBs
*G_IC_*/N·mm^−1^	4.9 ± 0.2	3.7 ± 0.2

**Table 7:** Parameters of the Young’s modulus ROM, regarding Adhesive A, *E_Adh_*, and the hollow glass beads, *E_GB_*.

Constituent	*E*/MPa
Adhesive A without GBs (prediction)	$1960\;\pm \;2\cdot \bar{\sigma }$
Hollow glass beads (prediction)	$5770\;\pm \;2\cdot \bar{\sigma }$

**Table 8:** Parameters of the Young’s modulus ROM, with regard to Adhesive B, *E_Adh_*, and the hollow glass beads, *E_GB_*.

Constituent	*E*/MPa
Adhesive B (*as supplied*)	$1862\;\pm \;2\cdot \bar{\sigma }$
Hollow glass beads (new prediction)	$2810\;\pm \;2\cdot \bar{\sigma }$

**Table 9:** Parameters of the *G_IC_*’s ROM, regarding Adhesive A, *G_Adh_*, and the hollow glass beads, *G_GB_*.

Constituent	*G_IC_/*N·mm^−1^
Adhesive A without GBs (prediction)	$3.2\;\pm \;2\cdot \bar{\sigma }$
Hollow glass beads (prediction)	$0.7\;\pm \;2\cdot \bar{\sigma }$

**Table 10:** Parameters of the *G_IC_*’s ROM, in regard to Adhesive B, *G_Adh_*, and the hollow glass beads, *G_GB_*.

Constituent	*G_IC_/*N·mm^−1^
Adhesive B (*as supplied*)	$4.9\;\pm \;2\cdot \bar{\sigma }$
Hollow glass beads (new prediction)	$1.2\;\pm \;2\cdot \bar{\sigma }$
